# Pneumomastia: A Rare but Benign Mammographic Finding

**DOI:** 10.5334/jbsr.3924

**Published:** 2025-06-24

**Authors:** Krucial Styslinger, Christina Cinelli

**Affiliations:** 1 Radiology Resident, MaineHealth Maine Medical Center, Portland, ME; 2 Radiologist, Spectrum Healthcare Partners, South Portland, ME

**Keywords:** pneumomastia, breast imaging, mammography

## Abstract

*Teaching point:* Pneumomastia is a rare but benign mammographic finding most often related to recent instrumentation. Typically, it does not require further workup, although it may necessitate repeat imaging when the finding is extensive enough to obscure small lesions or microcalcifications.

## Case History

**Figure 1:**
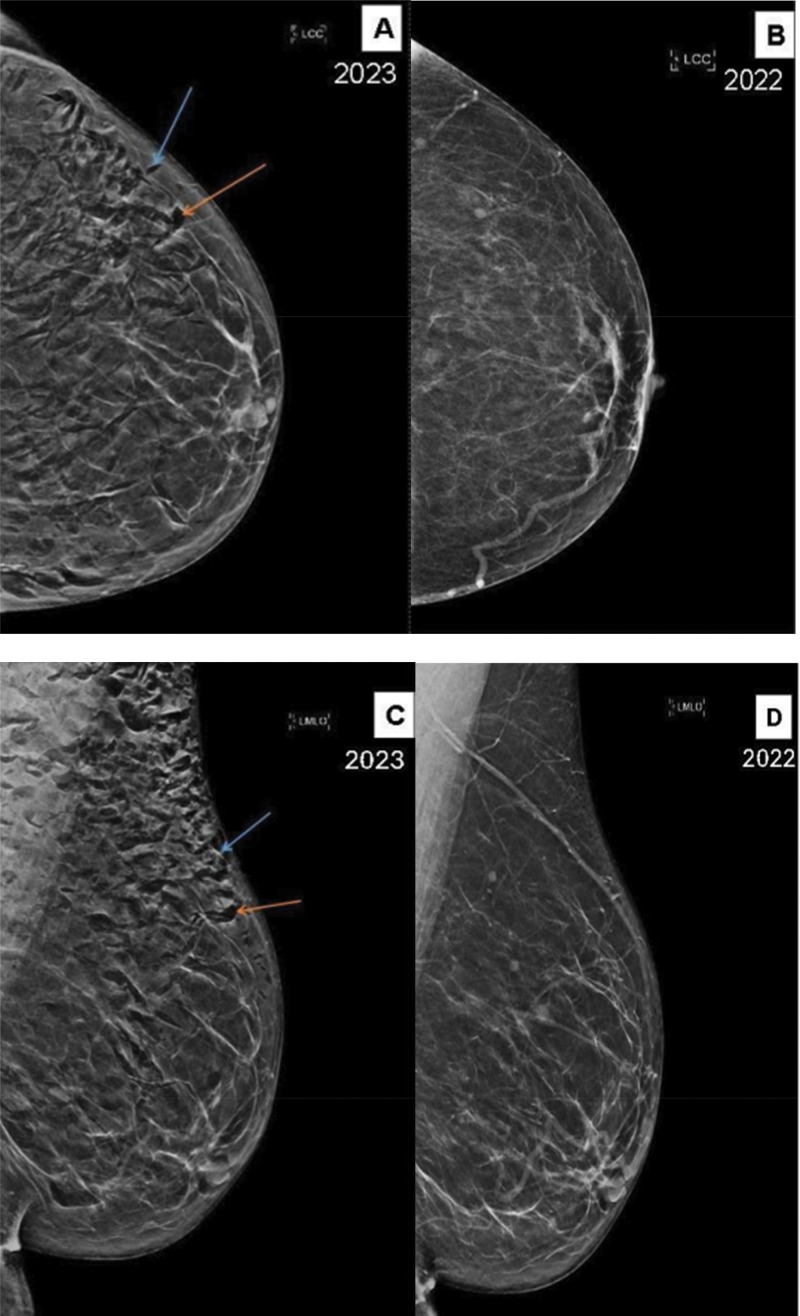
CC and MLO views of the left breast **(A, C)** showing extensive subcutaneous gas (orange arrows) as well as air outlining Cooper’s ligaments (blue arrows). These findings are new from the normal screening mammogram performed the year prior **(B, D)**.

A 57‑year‑old female with a history of obesity presented for a yearly screening mammogram. The physical examination was unremarkable. The left screening mammogram demonstrated extensive subcutaneous gas throughout the soft tissues of the left breast (orange arrows in Figures 1a, 1c). The air outlined Cooper’s ligaments as it tracked along them (blue arrows in Figures 1a, 1c). No air was seen within the ducts. These findings were not present on the mammogram performed the year before (Figures 1b, 1d). The right breast screening mammogram showed similar, if less extensive, findings of subcutaneous gas throughout the soft tissues of the right breast. In discussion with the patient, it was determined that the patient had undergone laparoscopic bariatric surgery several days prior. The findings and clinical history were consistent with pneumomastia, and the patient was returned to yearly screening.

## Comments

Pneumomastia is the presence of subcutaneous air within the breast. Although this is a benign finding, it is rare and is a potential cause of unnecessary workups in the absence of a thorough clinical history and the radiologist’s knowledge of this entity. It is most often caused by recent prior breast procedures, such as biopsy or breast augmentation. However, additional etiologies include recent laparoscopic surgery (due to high intraabdominal pressures from air insufflation). Anaerobic gas‑forming infection within the breast, bronchopleural fistula, recent thoracotomy, and spread of pneumomediastinum are less common etiologies for pneumomastia [1]. Given pneumomastia’s relation to recent instrumentation above or below the diaphragm, it is important to avoid scheduling breast imaging in the days immediately following a procedure.

When pneumomastia presents clinically, it can manifest as breast swelling with or without the presence of crepitus and typically patients are asymptomatic without physical exam findings of crepitus [1]. It is a self‑resolving process requiring conservative management when any of the above causes are elucidated through the patient history. On breast imaging, pneumomastia is apparent both mammographically and sonographically as subcutaneous gas within the superficial soft tissues tracking along Cooper’s ligaments. The absence of air within the ducts themselves is pertinent, as intraductal air is commonly seen in breastfeeding patients. Attention to breast density and the degree of pneumomastia is important, as in some extensive cases it could obscure small lesions and microcalcifications. In these cases, repeat imaging may be warranted. Correctly identifying pneumomastia as a benign finding can obviate unnecessary imaging workups as well as any associated costs and potential stress to the patient.
